# A High-Precision, Real-Time, and Robust Indoor Visible Light Positioning Method Based on Mean Shift Algorithm and Unscented Kalman Filter

**DOI:** 10.3390/s19051094

**Published:** 2019-03-04

**Authors:** Zekun Xie, Weipeng Guan, Jieheng Zheng, Xinjie Zhang, Shihuan Chen, Bangdong Chen

**Affiliations:** 1 School of Electronic and Information Engineering, South China University of Technology, Guangzhou 510640, China; xzkscut@163.com (Z.X.); eexinjie@mail.scut.edu.cn (X.Z.); eecbd_scut@mail.scut.edu.cn (B.C.); 2 School of Automation Science and Engineering, South China University of Technology, Guangzhou 510640, China; cnchensh@gmail.com; 3 School of Computer Science and Engineering, South China University of Technology, Guangzhou 510006, China; zjh_will@163.com

**Keywords:** visible light positioning (VLP), real-time positioning and tracking, image sensor (IS), mean shift (MS), unscented Kalman filter (UKF), robustness

## Abstract

Visible light positioning (VLP) is a promising technology for indoor navigation. However, most studies of VLP systems nowadays only focus on positioning accuracy, whereas robustness and real-time ability are often overlooked, which are all indispensable in actual VLP situations. Thus, we propose a novel VLP method based on mean shift (MS) algorithm and unscented Kalman filter (UKF) using image sensors as the positioning terminal and a Light Emitting Diode (LED) as the transmitting terminal. The main part of our VLP method is the MS algorithm, realizing high positioning accuracy with good robustness. Besides, UKF equips the mean shift algorithm with the capacity to track high-speed targets and improves the positioning accuracy when the LED is shielded. Moreover, a LED-ID (the identification of the LED) recognition algorithm proposed in our previous work was utilized to locate the LED in the initial frame, which also initialized MS and UKF. Furthermore, experiments showed that the positioning accuracy of our VLP algorithm was 0.42 cm, and the average processing time per frame was 24.93 ms. Also, even when half of the LED was shielded, the accuracy was maintained at 1.41 cm. All these data demonstrate that our proposed algorithm has excellent accuracy, strong robustness, and good real-time ability.

## 1. Introduction 

In business centers [1], large public buildings (subways, airports, libraries, etc.), high-risk industrial parks, hospitals, nursing homes [2], and other indoor places where Global Positioning System (GPS) helps little but where navigation and location services are urgently needed, indoor positioning technology has broad application prospects [3,4]. Common optional techniques for indoor positioning include infrared ray (IR), ultrasonic wave, radio-frequency identification (RFID), wireless local area network (WLAN), Bluetooth, ultra-wideband (UWB), etc. Based on these technologies, different implementation schemes have been developed. However, each has obvious drawbacks considering positioning accuracy [5] or the ability to resist electromagnetic interference [6], or the high cost of hardware devices [7], which makes them difficult to become popularized.

Compared with these indoor positioning technologies mentioned above, visible light positioning (VLP) technology has outstanding advantages because of its abundant bandwidth resources [8], strong ability to resist electromagnetic interference, higher positioning accuracy and illumination ability. Additionally, the cost of hardware devices supporting VLP systems is relatively small, because complex facilities conducting precise measurement are not necessary. A simple VLP system even can be structured with an image sensor, some LEDs and a processor. Therefore, VLP technology has a promising prospect in the field of indoor positioning. There exist two modes of indoor positioning systems based on VLP: The photodiode-based (PD-based) positioning, and the image-sensor-based (IS-based) positioning [9,10]. The PD-based VLP systems have been deeply studied by researchers before. A series of research projects on VLP systems based on PD have been completed in our previous works [11,12,13,14,15,16] through which we found that the mobility of the positioning terminal has been severely limited because of PD’s sensibility to the direction of the light beam. To deal with the orientations of the PDs, the authors of Reference [17] designed a pyramid receiver (PR) and hemispheric receiver (HR), which improved the channel capacity and bit-error-rate (BER) performance under various settings and reduced the space usage. However, another significant drawback of PD-based VLP systems is the doubtful robustness. Even at the same location, repeated measurements will yield a fluctuating value of light intensity. Also, PD-based VLP systems are easily disturbed by ambient light and reflected light [15]. Meanwhile, the angular and signal strength received need to be precisely measured. Otherwise the positioning results will have noticeable error. In addition, precise instruments must be exploited because the received signal strength and signal’s time difference of arrival should be measured to calculate the distance between the reference point and target [2]. In contrast, IS-based VLP methods are unaffected by these problems mentioned above. What is more, smart phones are nowadays equipped with high-resolution complementary metal-oxide-semiconductor (CMOS) sensor cameras, which can be combined with IS-based VLP methods easily, where huge commercial value lies. Furthermore, the position would fail if the LED is shielded in PD-based VLP systems. In comparison, our previous work [9] based on image sensor solved the problem effectively. Therefore, image sensors are better than PDs as the receiving terminal for VLP systems [18,19]. Finally, few research projects on PD-based VLP have explored the field of dynamic positioning, which has significance for real-time indoor navigation, but restricts the application to low-speed motion or static positioning [20]. 

Theoretically, IS has better performance than PD regarding the field of VLP, but existing studies have not yet yielded satisfactory results in terms of positioning accuracy, real-time ability, and robustness, which are three vital elements for VLP systems. There is still much room for improvement. First, for positioning accuracy, a circle projection-based single LED system was proposed in Reference [21], whose positioning accuracy was only 25.12 cm. In Reference [22], the positioning error was reduced to less than 10 cm; however, the performance remained unsatisfactory. These works did not give satisfactory accuracy. In Reference [23], a novel method was proposed utilizing the electronic compass and gyroscope to calculate the yaw angle of the positioning terminal. The stimulated results showed the error was within 2 cm. However, the real-time ability was not considered in the article. Additionally, few studies could maintain a good balance between real-time ability and positioning accuracy. The author used a minimax filter to estimate the trajectory of the terminal in Reference [24], whose average velocity simulated by MATLAB simulator was 1 m/s (3.6 km/h), unable to fit higher motion speed. Finally, the VLP method proposed in Reference [25] considers the system’s real-time ability, whose maximum allowable motion speed of the positioning terminal was 18 km/h, with a positioning accuracy of 7.5 cm. Though it maintained a relatively good balance between accuracy and real-time ability, the blur effect, caused by high relative speed between IS and LED, was ignored. When the target moves at such a speed, the image of the LED will blur. Because the IS-based positioning method in Reference [25] is based on pixel intensity detection, when the LED is blurred in the image, the LED–ID recognition process through pixel intensity detection becomes tough, which may lead to failure of location. The ignorance of robustness is crippling in the field of VLP, which can make research outputs have little practical value, and this reference article is no exception. Moreover, few papers have considered the general circumstance that the transmitter (i.e., LED) is shielded or broken. When the light links are blocked between LEDs and positioning terminal, the positioning would fail because most of the positioning algorithms are based on two or more LEDs, the absence of even one LED in the image can lead to failure of the whole algorithm. The shielded effect is a fatal problem in the field of VLP. No one has yet solved the problem except our previous work [9], where a relatively perfect method is proposed. Based on optical flow and Bayesian forecast, the algorithm possesses the ability to address all the difficulties of VLP systems mentioned above, whose positioning accuracy is 0.86 cm and the maximum allowable speed of positioning terminal is up to 48 km/h. Also, the algorithm can locate the target even if half of the LED is shielded. However, the computational cost of the optical flow method is very large. Furthermore, optical flow algorithms alone cannot track the LED under shielded effect. Therefore, Bayesian forecast is introduced to solve the problem of shielded effect. Finally, the outcomes of the Bayesian forecast algorithm and the optical flow algorithm was combined by Kalman filter to obtain the final output.

Though our previous work [9] seems powerful enough to address all the knotty problems in the field of VLP, it also raised some problems that cannot be ignored. First, the optical flow method used in Reference [9] greatly increases the computational cost and the running time of the whole system, which reduces the real-time ability of the algorithm, whose utility is inversely proportional to the computational cost. Because the function of the optical flow method is to track the LED, in this article, to improve the real-time ability of our visible light positioning method, it was replaced by the mean shift algorithm, which greatly reduces the computational cost. Moreover, the mean shift algorithm possesses wonderful robustness, which can reduce the influence of the shielded effect exists in the VLP situations to a great degree. Furthermore, in the simple background of VLP, the precision of the mean shift algorithm does not lose to any other target tracking algorithm. Second, the Kalman filter used in Reference [9] simply combines the outcome of optical flow and Bayesian forecast without considering the state noise and measurement noise of the whole system, which greatly affects the accuracy of the VLP system. In this article, the unscented Kalman filter is introduced to improve the robustness and accuracy of our visible light positioning method and process the noises of the whole VLP system in a clever way. 

In Reference [26], the author proved that the introduction of the unscented Kalman filter into the camshift algorithm can improve the accuracy of the target tracking algorithm as same as reducing the running time of the whole system by leaps and bounds. Inspired by Reference [26], our proposed algorithm also used unscented Kalman filter (UKF) to improve the performance of the mean shift algorithm, and eventually our VLP method. However, though the unscented Kalman Filter in Reference [26] took noises into account, the noises were only Gaussian white noises, which are random noises without considering the condition of the target. If the LED is not occluded and the measured position of the LED is accurate, the noises considered will reduce the accuracy. Therefore, inspired by Chaos theory, a weight measuring the accuracy of the measured LED’s position was introduced into our algorithm. If the accuracy of the measured LED’s position is reliable, the weight of Gaussian white noises will be small, and the measured position will be trusted more by the Kalman filter. Through this method, the bad influence of using Gaussian white noise as the state noise and measurement noise can be reduced.

To address the shortcomings of existing methods and improve the performance of our previous VLP system, in this paper, we propose a high-precision, real-time and robust indoor visible light positioning method based on the mean shift algorithm and unscented Kalman filter. The function of the LED is to deliver world coordinates using visible light. After the initial position of the positioning terminal is calculated by the LED–ID recognition algorithm, the mean shift algorithm is utilized to track and locate the LED in the image sequences in real time with high robustness. Then the trajectory of the positioning terminal can be calculated combining the positioning terminal’s initial position with its relative position relationship in the subsequent frames calculated by MS and UKF. The UKF algorithm forecasts the possible location of the LED in the next frame, equipping the mean shift algorithm with the ability to track fast moving targets and reduces the computation cost of the whole algorithm. Also, when the LED is shielded, the trajectory of the positioning terminal will be output by UKF to reduce the influence of noises. 

Previous studies have considerable drawbacks in robustness because they had to repeat the complex positioning algorithm to get the trajectory of the positioning terminal. However, in our VLP method, only the initial world position of the positioning terminal is calculated by the static positioning algorithm. The trajectory can be calculated using the relative position relationship between the LED and the positioning terminal in the image sequence, which can be accomplished by the target tracking algorithm. Most of the repeating process of the static positioning algorithm is replaced by robust and lightweight target tracking algorithm. The remainder of this paper is organized as follows: Section 2 provides a detailed description of the proposed positioning and tracking algorithm. The experimental setup and analysis are presented in Section 3. Eventually, we summarize our work in Section 4.

## 2. Theory

When our proposed method starts, the LED–ID recognition algorithm first finds the LED utilizing the image sensor, then the ID of the LED will be recognized. The details of the LED–ID recognition algorithm was deeply researched in our previous works [18,19]. 

**Figure 1:**
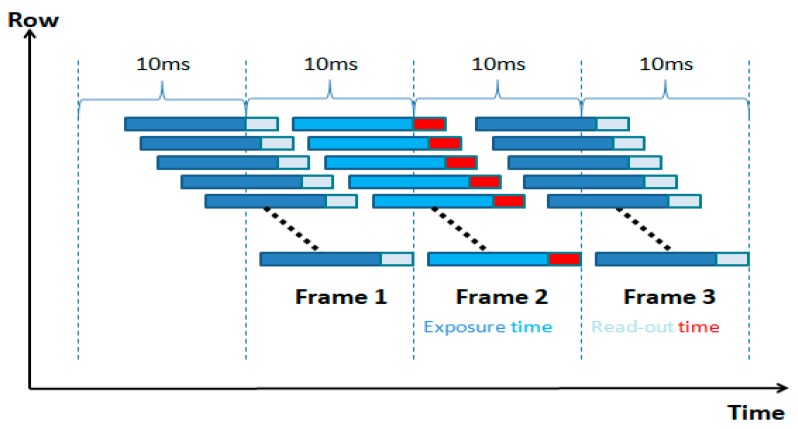
The working principle of the rolling shutter mechanism.

Our proposed method utilizes the rolling shutter mechanism of the Complementary Metal Oxide Semiconductor (CMOS) image sensor. The exposure and data readout are performed row by row. The data of one row read out immediately when the exposure of this row is finished. The working principle of the rolling shutter mechanism is shown in Figure 1. Instead of employing traditional LED–ID encoding and decoding methods, the process of LED–ID detection and recognition is regarded as a classifying problem regarding machine learning in our algorithm. Through off-line training for the classifiers and on-line recognition for LED–ID, high-speed and robust LED–ID recognition was realized. The LED–ID algorithm will not be detailed in this article. For readers interested in LED–ID recognition, please refer to our previous article [19]. 

After the LED–ID is recognized, the world’s coordinate of the LED is known. Then through single-light positioning technology, the initial real-world position of the positioning terminal can be obtained. Next, the mean shift algorithm and UKF dynamically track the LED in the image sequence, through which the relative position in the pixel coordinates between LED in the current frame and LED in the initial frame can be calculated. Utilizing different coordinate systems and the geometric relationships between the LED and the image sensor, linear mapping from the pixel coordinates to the world’s coordinates can be established. With the information above, the relative relationship between the LED’s current position and its initial frame in the pixel coordinate plane can be transformed to the relative position relationship in the world’s coordinate space. Finally, by combining the positioning terminal’s initial position with its relative position relationship in the subsequent frames, the location of the positioning terminal in the real world can be obtained. 

**Figure 2:**
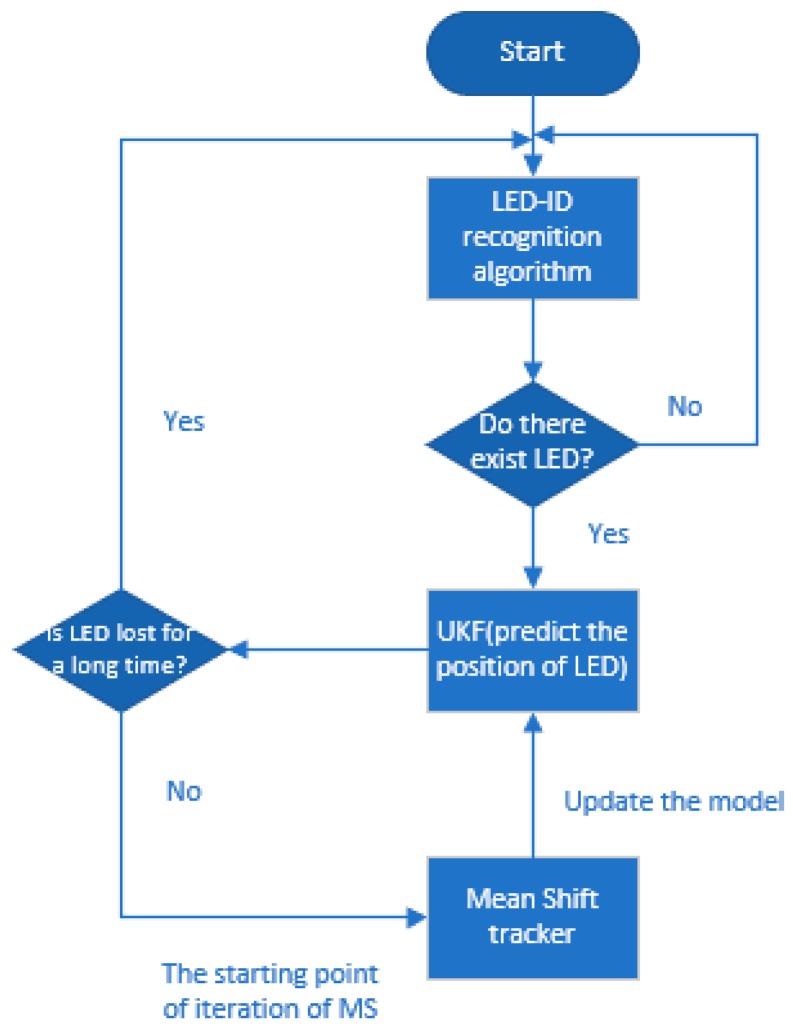
The flow of our proposed visible light positioning (VLP) algorithm.

Hence, by dynamically tracking the LED in the two-dimensional plane of the IS in the captured image sequences, indoor dynamic positioning can be accomplished. Because the processing time of our lightweight target tracking algorithm is much shorter than that of most static positioning systems, the real-time ability of our algorithm is also good. The steps of our whole algorithm are as follow: 

First, the LED–ID recognition algorithm obtains the LED’s initial world coordinates and pixel coordinates. The latter also initializes MS and UKF. Then the predicted position of UKF is treated as the starting position of iteration in MS. When ${\;c}_{b}$, the ratio of the target’s area factor $a_{p}$ to its initial value, is smaller than the threshold in 5 frames, which means LED is lost in the previous tracking process, the LED–ID recognition algorithm plays its role again to search for the initial position of the LED to start the next tracking cycle. After the LED is tracked and positioned in the pixel coordinate plane, the positioning terminal’s world coordinates can be calculated by the proposed algorithm. The flow of the whole algorithm is shown in Figure 2. In the following parts, these processes will be analyzed mathematically, and formulations will be given.

**Figure 3:**
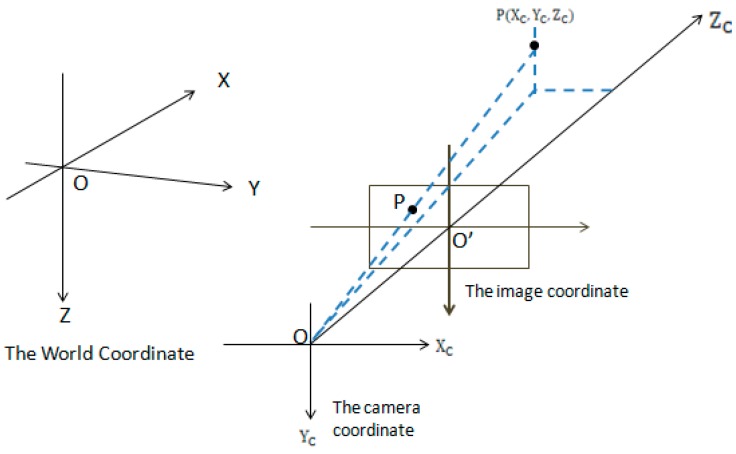
World coordinate system, image coordinate system, and camera coordinate system.

**Figure 4:**
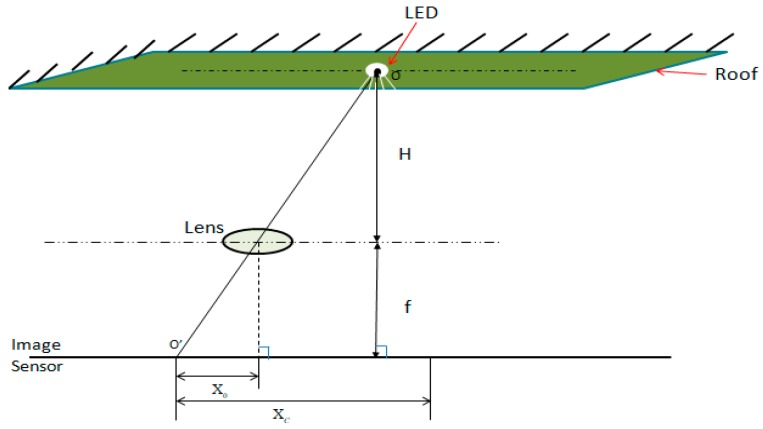
The geometrical relationship between the Light Emitting Diode (LED) and the image sensor.

After the procedures of LED–ID recognition, the initial position of the LED is obtained, which also initializes MS and UKF. Meanwhile, different coordinate systems, shown in Figure 3, and the geometrical relationship between the LED and image sensor, shown in Figure 4, are utilized to get the location of the positioning terminal in the real world. Define $L^{t}$ as the set of pixels belonging to LED at time t in the image, and ($x_{i}^{t},y_{i}^{t}$)$\in L^{t}$. i represents $i^{th}$ pixel. Because of the background’s area, such as the ceiling of the house, it is much larger than the LED’s area, and the position of the LED (s(t) ($x^{t},y^{t}$)) in the image can be approximated as the centroid of $L^{t}$.

The world’s coordinates of the image sensor is defined as $z_{l}$(t) ($x_{l}^{t}$,${\;y}_{l}^{t}$,$z_{l}^{t}$), where dx and dy represent each pixel’s size on IS in different coordinate directions, respectively. The height between the LED and lens is known as H, which is assumed as a fixed value and is already known by the algorithm. With all the conditions above, the pixel coordinate ($x_{0}$,$y_{0}$) of the image’s centroid can be calculated as follow:

(1) $$\left[\begin{array}{c} x_{0}\\ y_{0}\\ 1 \end{array}\right]=\left[\begin{array}{ccc} 1/\mathrm{dx} & 0 & x^{t}\\ 0 & 1/\mathrm{dy} & y^{t}\\ 0 & 0 & 1 \end{array}\right]\left[\begin{array}{c} x_{i}^{t}\\ y_{i}^{t}\\ 1 \end{array}\right]$$

Next, the horizontal coordinate of image sensor (camera) $X_{C}$ in the camera coordinate can be calculated by the following equation:

(2) $$\frac{X_{0}}{X_{C}}=\frac{f}{f+H}$$

**Figure 5:**
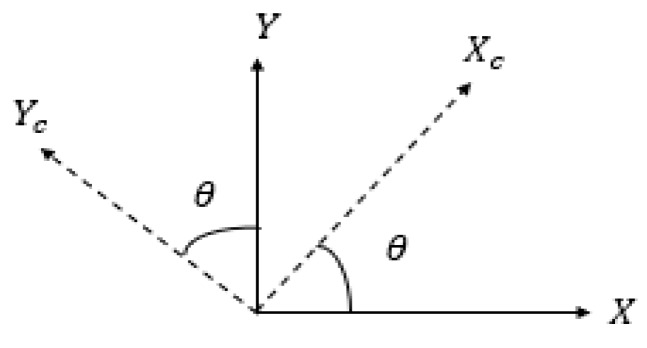
The transformation model of the camera coordinate system and the world’s coordinate system.

$Y_{C}$ can be calculated by the same method. As can be seen from Figure 5, we can rotate the coordinate by Equation (3) and realize positioning under any azimuth. $\left(x_{l}^{t},y_{l}^{t}\right)$ is transformed from $\left(X_{C},Y_{C}\right)$ by the equation below:

(3) $$\left[\begin{array}{c} X_{C}\\ Y_{C}\\ 1 \end{array}\right]=\left[\begin{array}{ccc} cos\theta & sin\theta & 0\\ -sin\theta & cos\theta & 0\\ 0 & 0 & 1 \end{array}\right]\left[\begin{array}{c} x_{l}^{t}\\ y_{l}^{t}\\ 1 \end{array}\right]+\left[\begin{array}{c} T_{X}\\ T_{Y}\\ T_{Z} \end{array}\right]$$

In the equation above,$\left[\begin{array}{c} T_{X}\\ T_{Y}\\ T_{Z} \end{array}\right]$ represents translation and ($x_{l}^{t}$,${\;y}_{l}^{t}$) has been denoted by $z_{l}$(t). Let $z_{l}$(t) = h(s(t),$D_{l}$), where h represents the mapping function and $D_{l}$ stands for the $i^{th}$ coordinate of the LED in the world’s coordinates that we have known.

### 2.1. The Mean Shift Algorithm

In some traditional VLP methods based on image recognition only, every image must be processed to locate the positioning terminal, where maximum allowable motion speed of the positioning terminal is confined by the blur effect caused by the high relative speed between LEDs and image sensors, whereas the blur effect has much less of an impact on the mean shift algorithm, relying on color histogram. Additionally, even if half of the LED is shielded in the image, the tracking process won't fail. In view of the wonderful robustness and real-time ability of the mean shift algorithm, it was used as the main part of our proposed VLP algorithm. Non-parametric density estimation is the basic concept in the mean shift algorithm, using kernel density estimation as the fundamental of the whole theory.

One of the most popular density estimation methods is known as kernel density estimation. Given n points of data ${\;X}_{i}$, i = 1…n in the d-dimensional space ${\;R}^{d}$, and the multivariate kernel density estimator whose kernel function K(x), together with a symmetric positive definite d × d bandwidth matrix H, calculated in the point x, satisfying:

(4) $$\hat{f}\left(x\right)=\frac{1}{n}\overset{n}{\underset{i=1}{\sum }}K_{H}\left(x-x_{i}\right)$$

in which

(5) $$K_{H}\left(x\right)={\left|H\right|}^{-\frac{1}{2}}K\left(H^{-\frac{1}{2}}x\right)$$

The kernel function K(x) with d variates is combined with the following equations:

(6) $$\int_{R^{d}}K\left(x\right)\mathrm{dx}=1\;$$

(7) $$\underset{\left|\left|x\right|\right|\to \infty }{\lim}{\left|\left|x\right|\right|}^{d}K\left(x\right)=0$$

(8) $$\int_{R^{d}}\mathrm{xK}\left(x\right)\mathrm{dx}=0\;$$

(9) $$\int_{R^{d}}{\mathrm{xx}}^{T}K\left(x\right)\mathrm{dx}=c_{K}I$$

where $c_{k}$ is a constant, and I is an Identity matrix. The multivariable kernel function can be generated by the following two methods.

(10) $$K^{P}\left(x\right)=\overset{d}{\underset{i=1}{\prod }}K_{1}\left(x_{i}\right)$$

(11) $$K^{S}\left(x\right)=a_{k,d}K_{1}\left(\left|\left|x\right|\right|\right)$$

in which $K^{P}\left(x\right)$ is obtained from the univariate kernels and $K^{S}\left(x\right)$ is the product of rotating $K_{1}$(x) in $R^{d}$. Besides, $K^{S}\left(x\right)$ is radially symmetric. The normalized constant $a_{k,d}$ assures that the integral of function $K^{S}\left(x\right)$ is one.

We only need to focus on a special class of kernel functions satisfying

(12) $$K\left(x\right)=c_{k,d}k\left({\left|\left|x\right|\right|}^{2}\right)$$

where k(x) is called the profile function of the K(x). The normalized constant $c_{k,d}$ is always positive, ensuring that the kernel function K(x) integrates to one.

H is a d × d bandwidth matrix which is often computed as diagonal H = diag[$h_{1}^{2}$,…$,h_{d}^{2}$], or proportional identity matrix H =$\;\;h^{2}$I. The proportional identity matrix has only one parameter, which can be computed more easily. If we use the equation H =${\;h}^{2}$I for calculation, then Equation (4) can be rewritten as

(13) $$\hat{f}\left(x\right)=\frac{1}{{\mathrm{nh}}^{d}}\overset{n}{\underset{i=1}{\sum }}K\left(\frac{x-x_{i}}{h}\right)$$

If the definition of one-dimension kernel function K(x) is brought into Equation (13), we can get the equation which the general mean shift algorithm uses to calculate the density estimate of eigenvalues.

The distribution position with maximum density in the sample data group can be obtained by estimating the density gradient. The density gradient is defined as the gradient of kernel density estimation function, which can be calculated by Equation (14):

(14) $$\begin{array}{ll} \hat{\nabla }f_{h,K}\left(x\right)=\nabla {\hat{f}}_{h,K}\left(x\right) & =\frac{2c_{k,d}}{{\mathrm{nh}}^{d+2}}\sum_{i=1}^{n}\left(x-x_{i}\right)k^{\prime }\left({\left|\left|\frac{x-x_{i}}{h}\right|\right|}^{2}\right)\\ & =\frac{2c_{k,d}}{{\mathrm{nh}}^{d+2}}\sum_{i=1}^{n}g\left({\left|\left|\frac{x-x_{i}}{h}\right|\right|}^{2}\right)\left(\frac{\sum_{i=1}^{n}x_{i}g{\left(\left|\left|\frac{x-x_{i}}{h}\right|\right|\right)}^{2}}{\sum_{i=1}^{n}g{\left(\left|\left|\frac{x-x_{i}}{h}\right|\right|\right)}^{2}}-x\right)\\ & =f_{h,G}\left(x\right)m_{h,G}\left(x\right) \end{array}$$

in which $f_{h,G}$(x) is the non-parametric density estimation function based on kernel G(x) at point x, and $m_{h,G}$(x) is a mean shift vector. Besides, g(x) = −$k^{\prime }\left(x\right)$ is the profile function of kernel G(x).

In general, the shorter the distance of the sample point to the central point, the more significant the statistical property of the estimated point x becomes. Thus, the concept of kernel density function is introduced, giving each sample point a different weight associating with their distance to the center point. Also, the mean shift procedure is guaranteed to make the kernel function converge at a nearby point where the estimate density gradient is zero.

Epanechikov kernel function is used for model description. In initial frame, supposing that there are n pixels ${\left\{x_{i}\right\}}_{i=1,\ldots n}$ in the target region, and the center point is $x_{0}$. If the bandwidth of the kernel function is h, and we uniformly divide the feature space into m subintervals, the probability density estimation of the eigenvalue of the target model u = 1,…,m is

(15) $${\hat{p}}_{u}\left(y\right)=C\overset{n}{\underset{i=1}{\sum }}k\left({\left|\left|\frac{x_{0}-x_{i}}{h}\right|\right|}^{2}\right)\delta \left(b\left(x_{i}\right)-u\right)$$

(16) $$C=\frac{1}{\sum_{i=1}^{n}k\left({\left|\left|\frac{x_{0}-x_{i}}{h}\right|\right|}^{2}\right)}$$

where C is a normalization constant, and function k() is the profile function of kernel, measuring the weight of each pixel by the distance from which to the center point $x_{0}$, as stated above. δ(b($x_{i}$) − u) judges whether the eigenvalue of pixel $x_{i}$ belongs to the $u^{th}$bin. The candidate regions in the subsequent frames are described in the same way.

Because of the excellent fitness between Bhattacharyya coefficient and the mean shift algorithm, the Bhattacharyya coefficient is selected to measure the similarity between target model and candidate model in our proposed algorithm.

The eigenvalue of each model in the feature space is divided into m parts. If public members of two models in a part can be found when the Bhattacharyya coefficient is being computed in each part, the value of Bhattacharyya coefficient grows. The value of m depends on the range of eigenvalue in the feature space. The accuracy of the Bhattacharyya coefficient will be influenced whether m is too large or too small. Here is the definition of Bhattacharyya coefficient:

(17) $$\rho \left(y\right)=\rho \left(\hat{p}\left(y\right),\hat{q}\right)=\overset{m}{\underset{u=1}{\sum }}\sqrt{{\hat{p}}_{u}\left(y\right){\hat{q}}_{u}}$$

where ρ(y) ∈ [0,1], whose value represents the similarity between the two models. The candidate region that make the maximum value of ρ(y) is believed to be the position of the target. 

The value of Bhattacharyya coefficient ρ(y) should be maximized if we want to get the most accurate position of the target. Normally, the center point $y_{0}$ of the target in last frame is treated as the initial position of the target in current frame, where the algorithm begins searching for the goal of optimal matching, whose center point is y. Firstly, calculate the probability density estimation ${\hat{p}}_{u}\left(y_{0}\right)$ of the candidate target at point $y_{0}$ in the current frame, the current Bhattacharyya coefficient satisfying:

(18) $$\rho \left(y_{0}\right)=\overset{m}{\underset{u=1}{\sum }}\sqrt{{\hat{p}}_{u}\left(y_{0}\right){\hat{q}}_{u}}$$

whose Taylor expansion is computed by:

(19) $$\rho \left(y_{0}\right)=\overset{m}{\underset{u=1}{\sum }}\sqrt{{\hat{p}}_{u}\left(y_{0}\right){\hat{q}}_{u}}=\frac{1}{2}\overset{m}{\underset{u=1}{\sum }}\sqrt{{\hat{p}}_{u}\left(y_{0}\right){\hat{q}}_{u}}+\frac{C_{h}}{2}\overset{n_{k}}{\underset{i=1}{\sum }}w_{i}k\left({\left|\left|\frac{y-x_{i}}{h}\right|\right|}^{2}\right)$$

in which $w_{i}$ is the weight of each pixel, whose definition is:

(20) $$w_{i}=\overset{m}{\underset{u=1}{\sum }}\sqrt{\frac{{\hat{q}}_{u}}{{\hat{p}}_{u}\left(y_{0}\right)}}\delta \left(b\left(x_{i}-u\right)\right)$$

It is easy to notice that only the second term of Equation (19) is associated with y. If it gets the maximum value, the value of Bhattacharyya coefficient is maximized too.

Thus, we analyze the second term, to define:

(21) $$f_{n,K}=\frac{C_{h}}{2}\overset{n_{k}}{\underset{i=1}{\sum }}w_{i}k\left({\left|\left|\frac{y-x_{i}}{h}\right|\right|}^{2}\right)$$

which is very similar with the definition of the kernel density function. The mean shift vector also can be calculated, pointing towards the center point y of the target’s actual position from the candidate point ${\;y}_{0}$, satisfying:

(22) $$m_{h,G}\left(y\right)=y-y_{0}=\frac{\sum_{i=1}^{n_{k}}x_{i}w_{i}g\left({\left|\left|\frac{y-x_{i}}{h}\right|\right|}^{2}\right)}{\sum_{i=1}^{n_{k}}w_{i}g\left({\left|\left|\frac{y-x_{i}}{h}\right|\right|}^{2}\right)}-y_{0}$$

**Figure 6:**
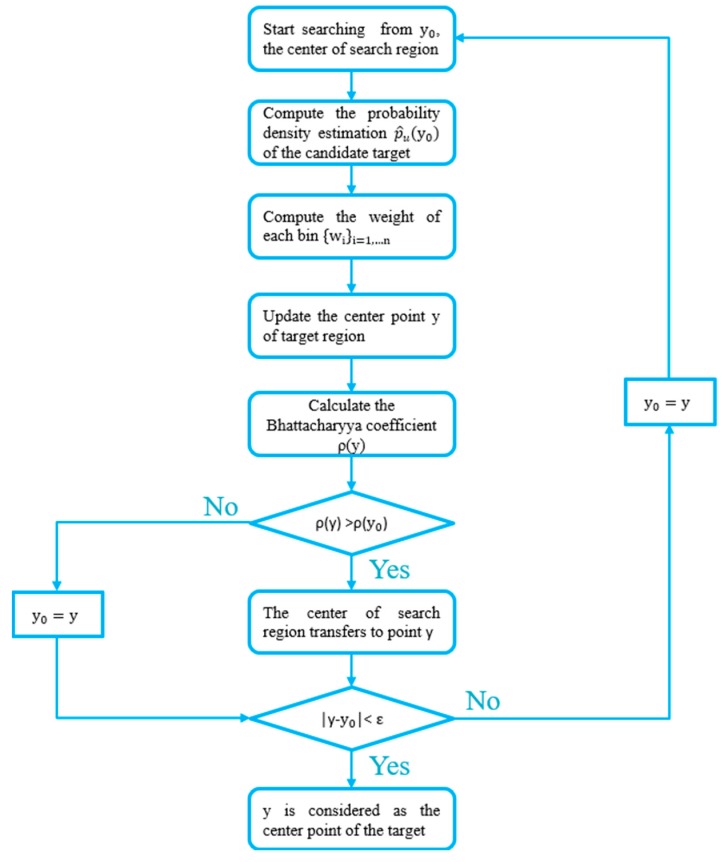
The process of the mean shift algorithm.

Supposing that the distribution of target’s model is ${\hat{q}}_{u}$ u = 1,…,m, the estimated position in the current frame is $y_{0}$, and the error allowed is ε, then the mean shift algorithm can be implement by the following steps, as is shown in Figure 6:

1. Assume that the target’s initial position in the current frame is the central point $y_{0}$ of the target in last frame. Firstly, compute the probability density estimation ${\hat{p}}_{u}\left(y_{0}\right)$ of the candidate target in current image frame using the same method as Equation (15). Then, utilize Equation (17) to calculate the Bhattacharyya coefficient ρ($y_{0}$);
2. Compute the weight of each bin ${\left\{w_{i}\right\}}_{i=1,\ldots n}$ with Equation (20);
3. Update the center point y of target region;
4. Calculate the Bhattacharyya coefficient ρ(y);
5. If ρ(y) > ρ($y_{0}$), the center of the search region transfers to point y;
6. If |y − $y_{0}$| < ε, the point y is considered as the center point of the target region in the current frame, else skip to step 1.

### 2.2. The Unscented Kalman Filter

Though the traditional mean shift algorithm possesses excellent real-time ability and accuracy under ideal situations, it cannot track targets with high speed because the algorithm starts searching the candidate target from its central point in the last frame within a small area. If the target moves outside the area within the time of one frame, the algorithm fails. To improve the real-time ability and the robustness of our proposed algorithm, giving it the ability to track objects moving at a high speed as well as to pinpoint the target under shielding effect, we introduced the unscented Kalman filter into our detection algorithm.

**Figure 7:**
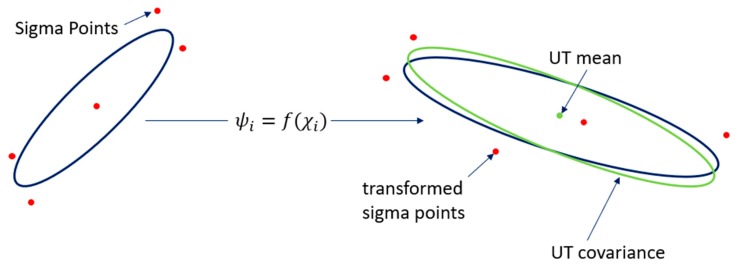
The process of unscented transformation.

**Figure 8:**
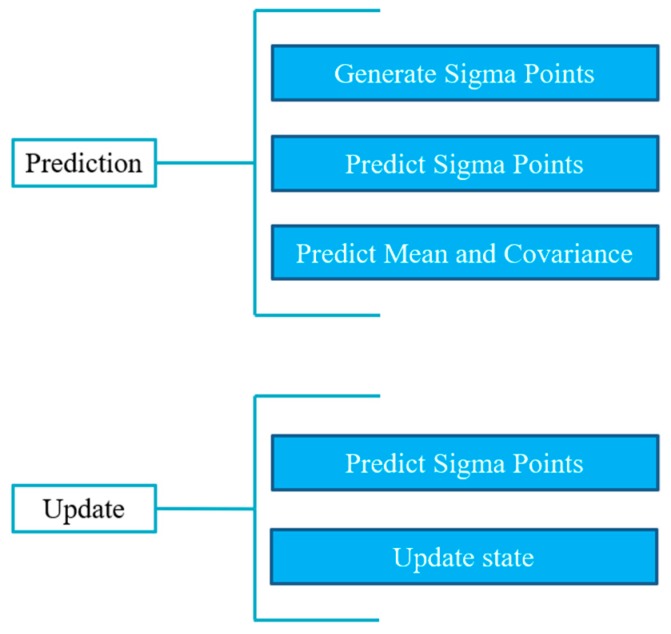
The process of unscented Kalman filter.

Based on the unscented transformation (UT), shown in Figure 7, UKF abandons the traditional method of linearization of non-linear functions and adopts the framework of the Kalman filter, which is shown in Figure 8. On the premise that the mean value and covariance of the random vectors remain unchanged, a set of Sigma sample points was selected, each of which goes through non-linear transformations. Then the mean and variance of the random vector through the non-linear transformation were estimated by the statistics of the transformed sample points, avoiding the error caused by linearization. The UKF algorithm has better stability than EKF because it no longer calculates the Jacobi matrix of non-linear equations. Also, UKF has a similar performance and smaller calculation cost compared with particle filter.

An ordinary state space model can be divided into two parts: namely state transfer model and state observation model. The state of the tracking system is the state of the target, while the observation model is the sequence image. Given that the velocity of the target changes, we assume the acceleration a(k) is a random quantity, and a(k) satisfying the Gauss distribution a(k) ∝ $N(0,\sigma_{w}^{2})$. The target’s state vector was set to be $X\left(k\right)={\left[x,y,\dot{x},\dot{y},c_{b}\right]}^{T}{}_{k}$, where (x, y) was the target’s center point; $\dot{x}$ and $\dot{y}$ represent the velocity of the target at the x and y coordinate directions, respectively; and $c_{b}$ was the ratio of the target’s current area in the tracking window to its initial area. Observation variable $\;y\left(k\right)={\left[x_{c}\left(k\right),y_{c}\left(k\right)\right]}_{k}^{T}$, in which $x_{c}\left(k\right)$ and $y_{c}\left(k\right)$ are observation values of the target. The state transition model and observation model are respectively computed by:

(23) $$X\left(k\right)=\phi X\left(k-1\right)+{\Gamma w}_{k}$$

(24) $$y\left(k\right)=X_{s}\left(k\right)+{\Xi v}_{k}$$

where $\phi =\left[1,0,t,0,0;0,1,0,t,0;0,0,1,0,0;0,0,0,1,0;0,0,0,0,a_{p}\right];\Gamma ={\left[\frac{t^{2}}{2},\frac{t^{2}}{2},t,t,0\right]}^{T};\;\Xi ={\left[1,1\right]}^{T};$ $X_{s}\left(k\right)={\left[x_{m}\left(k\right),y_{m}\left(k\right)\right]}_{.}^{T}\;w_{k}$, and $v_{k}$ represent the value of white Gaussian noise of the state transition model and observation model, respectively. t is the time interval of adjacent frames, which is one in our algorithm. $a_{p}$ is the target’s area factor, which is computed by the zeroth moment of the image in the tracking window. $(x_{m}$(k),$y_{m}\left(k\right))$ is the centroid of the LED obtained from the mean shift algorithm. Supposing that $(x_{c}$,$y_{c})$ is the initial value of the LED’s centroid, calculated by our LED–ID detection algorithm, X(0) =$\;\;{\left[x_{c},{\;y}_{c},\;0,\;0,\;1\right]}^{T}$. The theory and formula of the unscented Kalman filter is shown below.

Assume that state’s mean and variance of the n-dimension state vector X at time k − 1 are ${\hat{x}}_{k-1}$ and P(k − 1) respectively. The state transition model and observation model are:

(25) $$x_{k}=F\left(x_{k-1}\right)+W_{k}$$

(26) $$y_{k}=H\left(x_{k}\right)+V_{k}$$

where F is the state transition equation and H is the observation equation (specially,$\;H\left(x_{k}\right)=X_{s}\left(k\right)$ in our algorithm). ${\;W}_{k}$ and $V_{k}$ are the white Gaussian noise matrices of these two models (in our algorithm $W_{k}={\Gamma w}_{k}$ and $V_{k}={\Xi v}_{k})$, whose statistical characteristics satisfy:

(27) $$W_{k}\sim N\left(0,Q_{k}\right)\;\;\;V_{k}\sim \;N\left(0,R_{k}\right)$$

in which $Q_{k}$ and $R_{k}$ are the covariance matrix of two noise matrices, respectively. 

(1) Initialization of UKF

(28) $${\hat{x}}_{0}=E\left[x_{0}\right]$$

(29) $$P_{0}=E\left[\left(x_{0}-{\hat{x}}_{0}\right){\left(x_{0}-{\hat{x}}_{0}\right)}^{T}\right]$$

The Sigma points can be calculated by Equations (30)–(32).

(30) $$\chi_{k-1}^{0}={\hat{x}}_{k-1}$$

(31) $$\chi_{k-1}^{i}={\hat{x}}_{k-1}+{\left(\sqrt{\left(n+\lambda \right)P_{k-1}}\right)}_{i},i=1,\cdots ,n$$

(32) $$\chi_{k-1}^{i}={\hat{x}}_{k-1}-{\left(\sqrt{\left(n+\lambda \right)P_{k-1}}\right)}_{i},i=n+1,\cdots ,2n$$

where $\lambda =\alpha^{2}\left(n+\varphi \right)-n;\varphi =3-n,\;\alpha \;$ is the candidate parameter, and $0<\alpha \leq 10^{-4}$.

(2) Time updating process. Take Sigma points into the state transition Equation (33) and observation Equation (36), then compute the state vector’s average value by Equation (37)

(33) $$\chi_{k|k-1}^{i}=F\left(\chi_{k-1}^{i}\right)$$

(34) $${\hat{x}}_{k}^{-}=\sum_{i=0}^{2n}W_{i}^{\left(m\right)}\chi_{k|k-1}^{i}$$

(35) $$P_{x,k}^{-}=\overset{2n}{\underset{i=0}{\sum }}W_{i}^{\left(c\right)}\left[\chi_{k|k-1}^{i}-{\hat{x}}_{k}^{-}\right]{\left[\chi_{k|k-1}^{i}-{\hat{x}}_{k}^{-}\right]}^{T}+Q_{k}$$

(36) $$\gamma_{k|k-1}^{i}=H\left(\chi_{k|k-1}^{i}\right)$$

(37) $${\hat{y}}_{k}^{-}=\sum_{i=0}^{2n}W_{i}^{\left(m\right)}\gamma_{k|k-1}^{i}$$

where $W_{i}^{\left(m\right)}$ is the weight coefficient of the mean. $W_{0}^{\left(m\right)}=\frac{\lambda }{n}+\lambda$; $W_{i}^{\left(m\right)}=1/\left[2\left(n+\lambda \right)\right]$, i = 1,…,2n. 

(3) Observation updating equations. Take Equations (34), (36), (37) into Equations (38) and (39), compute Kalman gain by Equation (40).

(38) $$P_{y,k}=\overset{2n}{\underset{i=0}{\sum }}W_{i}^{\left(c\right)}\left[\gamma_{k|k-1}^{i}-{\hat{y}}_{k}^{-}\right]{\left[\gamma_{k|k-1}^{i}-{\hat{y}}_{k}^{-}\right]}^{T}+R_{k}$$

(39) $$P_{\mathrm{xy},k}=\overset{2n}{\underset{i=0}{\sum }}W_{i}^{\left(c\right)}\left[\chi_{k|k-1}^{i}-{\hat{x}}_{k}^{-}\right]{\left[\gamma_{k|k-1}^{i}-{\hat{y}}_{k}^{-}\right]}^{T}$$

(40) $$K=P_{\mathrm{xy},k}P_{y,k}^{-1}$$

in which $W_{0}^{\left(c\right)}=\lambda /\left(n+\lambda \right)$+ (1 − $\alpha^{2}+\beta$); $W_{i}^{\left(c\right)}=W_{i}^{\left(m\right)}\left(i=1,\ldots 2n\right);\;\beta \geq$0 and the value of β is 0 here.

Particularly in our algorithm, a confidence coefficient ω is introduced into Equation (38), which can be rewritten as:

(41) $$P_{y,k}=\overset{2n}{\underset{i=0}{\sum }}W_{i}^{\left(c\right)}\left[\gamma_{k|k-1}^{i}-{\hat{y}}_{k}^{-}\right]{\left[\gamma_{k|k-1}^{i}-{\hat{y}}_{k}^{-}\right]}^{T}+{\omega R}_{k}$$

If there exists little interference or shielded area of the LED in the image, in other words, $c_{b}\approx 1$, which means $(x_{m}$(k),$y_{m}\left(k\right))$ can been viewed as the real centroid of the LED, let ω≈0 to reduce the influence of the noise matrix $R_{k}$, meaning the original observation value is trusted. However, if the value of $c_{b}$ is close to 0, which means most areas of the LED are occluded in the image, the outcome of MS cannot be trusted. Because the centroid of the candidate region will be viewed as the actual centroid of the LED by the mean shift algorithm, whose error is considerable. In this condition, it is necessary to give the noise matrix a bigger weight. Originally, $c_{b}\in \left[0,1\right]$. To obtain proper weight, $c_{b}$ will be normalized into [−2ℵ,ℵ], where ℵ=1 in our algorithm. Thus, $\omega \in \left[10^{-2ℵ},10^{ℵ}\right]$. When $c_{b}\approx 1$, $\omega \approx 10^{-2}$ in our algorithm. 

The mean and variance of the state vector can then be updated after taking Kalman gain into Equations (42) and (43):

(42) $${\hat{x}}_{k}={\hat{x}}_{k}^{-}+K\left(y_{k}-{\hat{y}}_{k}^{-}\right)$$

(43) $$P_{x,k}=P_{y,k}^{-}-{\mathrm{KP}}_{y,k}K^{T}$$

Due to the uncertainty of moving targets and model, each time the target’s position calculated by our proposed algorithm will be compared with its last position to update and correct the state model of unscented Kalman filter.

Through introducing UKF into the mean shift algorithm, our proposed algorithm possesses the capacity to track fast moving targets because the mean shift algorithm starts searching for the candidate target from the position predicted by UKF (${\hat{x}}_{k}^{-}$, the prediction value of state equation in Equation (27)) in current frame instead of from the centroid of the target in last frame, which also reduces the number of iteration of MS. The candidate search region in the current frame was chosen reasonably by UKF, taking the priori positions of the target into consideration, thus the problem of the target’s giant velocity making it move outside the candidate search region within the time of one frame has been solved. Meanwhile, because of the reduced average iteration number, the real-time ability of the algorithm was enhanced too. When the target was located, its current position was compared with previous positions to estimate its velocity and the most possible position in the next frame, through which the state model of UKF was updated. The combination of UKF and the mean shift algorithm makes a closed loop tracking system which can track fast moving targets in real-time effectively.

## 3. Experiment and Result

### 3.1. Experimental Facilities 

**Figure 9:**
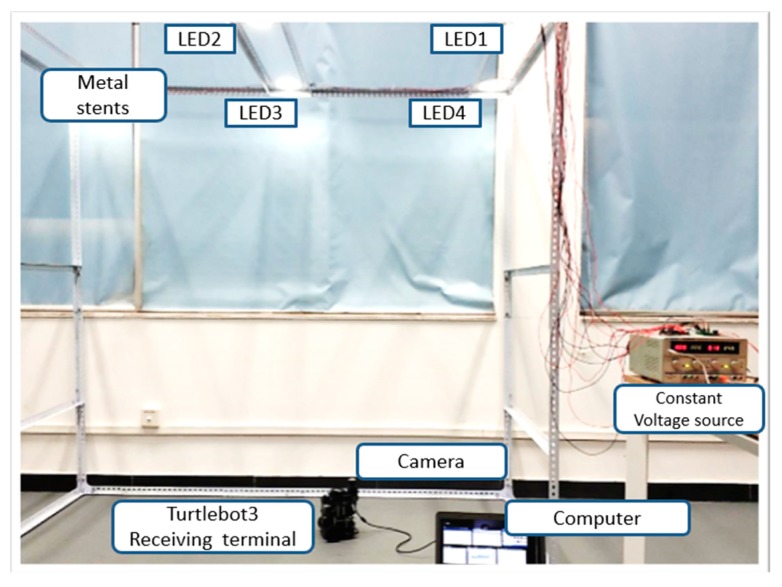
The platform and hardware devices for experimental setup.

Our experimental facilities are shown in Figure 9, including a constant voltage source, turlebot3 robot, an industry camera, 4 LEDs, a personal computer (ThinkPad E475, Windows 10, 4G RAM A10-9600P CPU@2.4GHz, Lenovo, Beijing, China.), and a high-speed video transmission line. The LEDs were light signal transmitters with unique IDs, supplied with a constant voltage source. The turlebot3 robot served as the carrier for the industry camera, which can move along a fixed route if a script is written beforehand. The industry camera was connected to the PC by a long and high-speed video transmission line. The time of transmission was included in the processing time of our VLP system. The combination of turlebot3 and industry camera stimulated the moving object requiring indoor navigation. The personal computer received the image captured by the industry camera and processed it with our proposed algorithm in real time. 

The size of our platform was 190 cm×100×190 cm. Four of the LEDs were used to realize the VLP system, whose world coordinates were (100,145,190), (0,145,190), (0,45,190) and (100,45,190), respectively. The specific parameters of the industry camera, turlebot3, constant voltage source, and experiment platform are shown in Table 1. 

The open source computer vision library (Opencv2.4.9) was used to process the received images in our experiment, and C++ was used as the programming language. Also, we recorded several image sequences in different situations with the same route of turlebot3 to better analyze the performance of our proposed algorithm, which were all captured by the industry camera mentioned above. The route of turlebot3 was constant, realized by a script written beforehand. Moreover, the single-lamp positioning technology was introduced into our VLP systems, meaning that we did not need to acquire all the LEDs’ coordinates but just one to locate the target, which reduced the computational cost and enhanced the real-time ability of our VLP algorithm.

Furthermore, to learn the ground truth reference of the robot’s position, another camera was set directly above the experimental platform. Grids and coordinates were drawn on the ground before doing the experiment. When the industry camera on the turtlebot3 started working, the camera above the experimental platform started recording. The videos captured by the two cameras were one-to-one in the number of frames. The frames with the same number were taken at the same time. When we wanted to confirm the ground truth position of the turtlebot3 at a certain frame, we only needed to record the number of this frame and then find the frame with the same number in the videos recorded by the camera above the platform. Finally, we read the coordinates from the grids on the ground where the turtlebot3 stood from the image of this frame. Through this method, we learned the ground truth reference of the turtlebot3’s position, which can be utilized to measure the positioning accuracy of our VLP system.

### 3.2. Result and Analysis 

**Figure 10:**
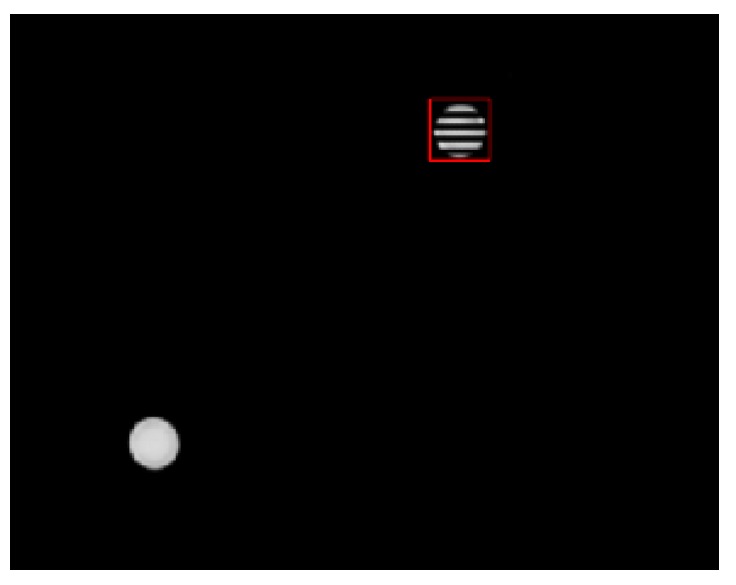
The tracking performance when the LED was not shielded.

Positioning accuracy is a vital index measuring the performance of VLP systems. Though the LED may be shielded or broken sometimes, in most cases the whole LED is captured by the positioning terminal. Besides, an interferential lamp was introduced into our experiment to test if our LED–ID algorithm could successfully detect the position of the LED and obtain its ID with the world’s coordinates. The result was successful as shown in Figure 10.

**Figure 11:**
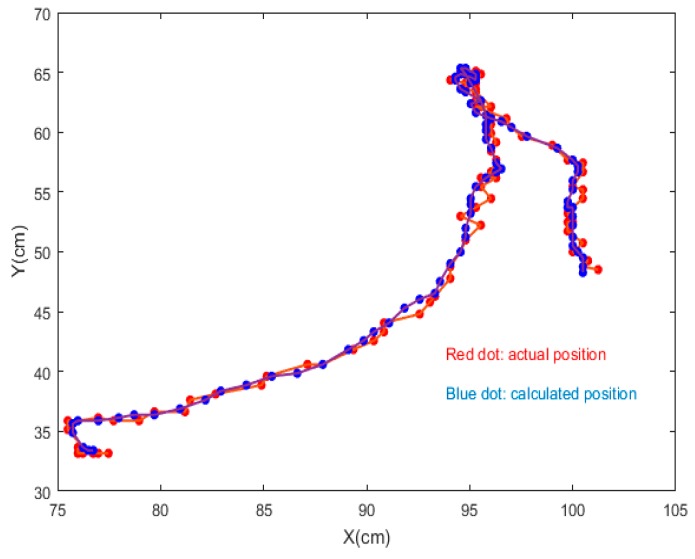
The calculated positioning value and actual positioning value of the positioning terminal.

Thus, 87 sequential frames without shielding were chosen to measure the positioning accuracy of the proposed algorithm in our experiment. The result is shown in Figure 11 where the red dot stands for the actual position and the blue dot represents the results calculated by our algorithm. From Figure 11 we can learn that our proposed algorithm has high accuracy directly.

**Figure 12:**
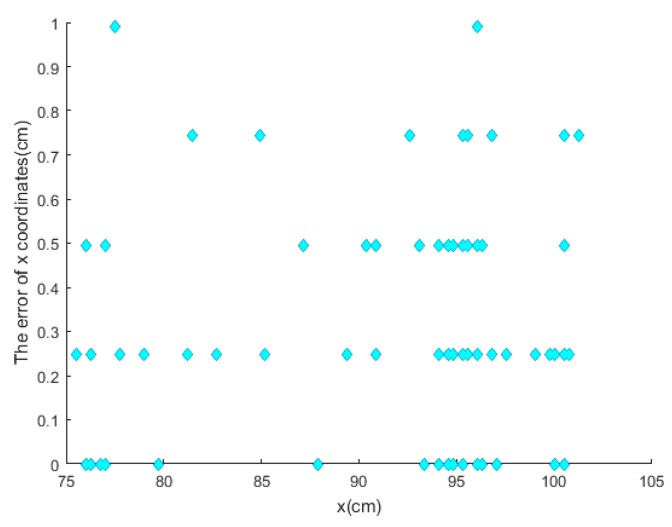
The error of position value of x coordinates.

**Figure 13:**
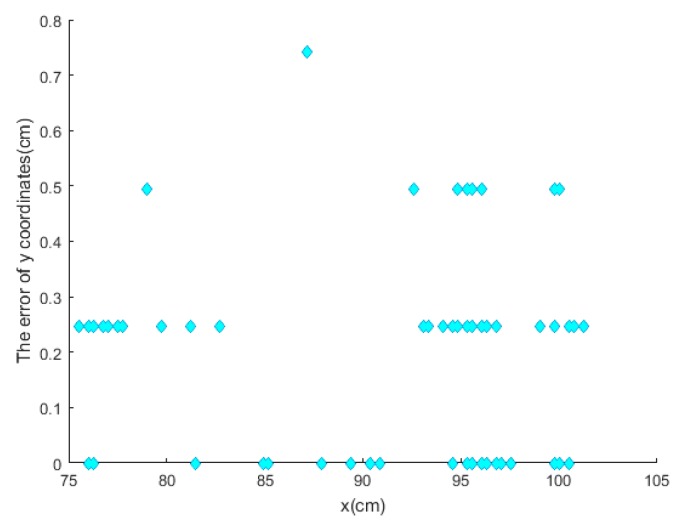
The error of position value of y coordinates.

**Figure 14:**
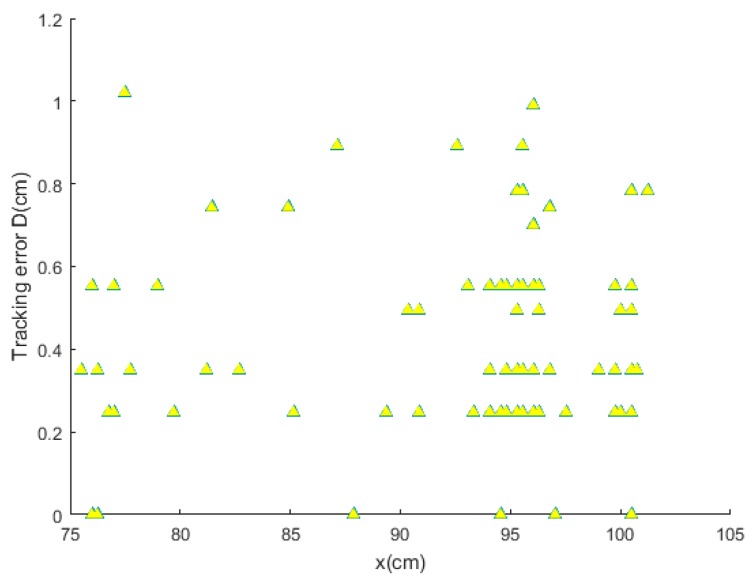
The tracking error D.

For more accurate analysis, the positioning error of the x and y coordinates and the tracking error D were further analyzed, respectively, in Figure 12, Figure 13, and Figure 14.

We define positioning error as:

(44) $$D=\sqrt{{\left(X-X_{r}\right)}^{2}+{\left(Y-Y_{r}\right)}^{2}}$$

(45) $$\mathrm{Error}\;\mathrm{of}\;X(Y)\;=\;|{\mathrm{Value}}_{\mathrm{predicted}}-{\mathrm{Value}}_{\mathrm{actual}}|$$

where (X, Y) is the position located by our proposed algorithm and ($X_{r},Y_{r}$) is the actual position. The maximal positioning error of x coordinates is less than 1 cm, and that of y coordinates less than 0.8 cm. Also, the value of maximal positioning error does not exceed 1.2 cm. Besides, the average value of positioning error in x coordinates, the average value of error in y coordinates, the average positioning error were 0.31 cm, 0.21 cm, and 0.42 cm, respectively.

Cumulative distribution function (CDF) is the integral of the probability density function, which can be used to analyze the probability distribution of the tracking error, error in the direction of coordinate axis x and coordinate axis y.

The cumulative distribution function of positioning error is shown in Figure 14. The definition of cumulative distribution function is:

(46) $$F_{X}\left(x\right)=P\left(X\leq x\right)$$

In other words, the cumulative distribution function represents the sum of the probability of all values less than or equal to x for the discrete variable.

**Figure 15:**
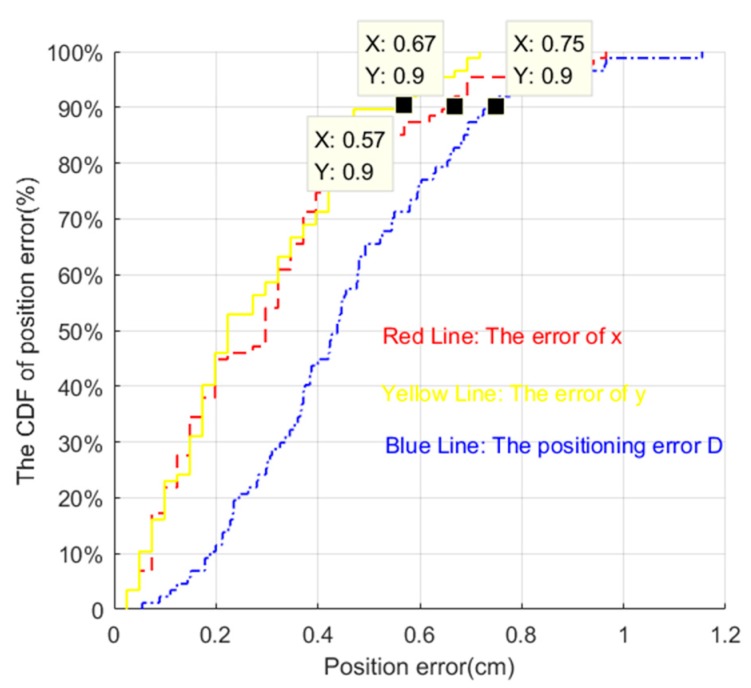
The cumulative distribution function (CDF) of positioning error.

As the Figure 15 indicates, more than 90% of positioning error D, the positioning error of x coordinates, and the positioning error of y coordinates were less than 0.75 cm, 0.67 cm, and 0.57 cm, respectively. If we can bear the 10% uncertainty, the positioning error of our algorithm is only 0.75 cm. In contrast, the positioning error of our previous work [9] was two times more than that of our new method.

The real-time ability is also important for VLP systems, which determines the maximum motion speed of the positioning terminal allowed. The running time and complexity of the algorithm both affect the real-time ability of VLP algorithms prominently. Because the algorithm needs time to process one frame, during which the target may already move to other positions. The process of positioning is always slower than the moving of the target. Our proposed algorithm utilizes the unscented Kalman filter algorithm to predict the most possible position of the target in the current frame. Then let the mean shift algorithm search for the target from the predicted position. This method increases the highest allowable motion speed of the positioning terminal. Theoretically, our proposed algorithm can track targets with any speed if enough priori information is given. However, if the target moves too quickly within one frame, the LED will exceed the maximum capture scope of the image sensor.

**Figure 16:**
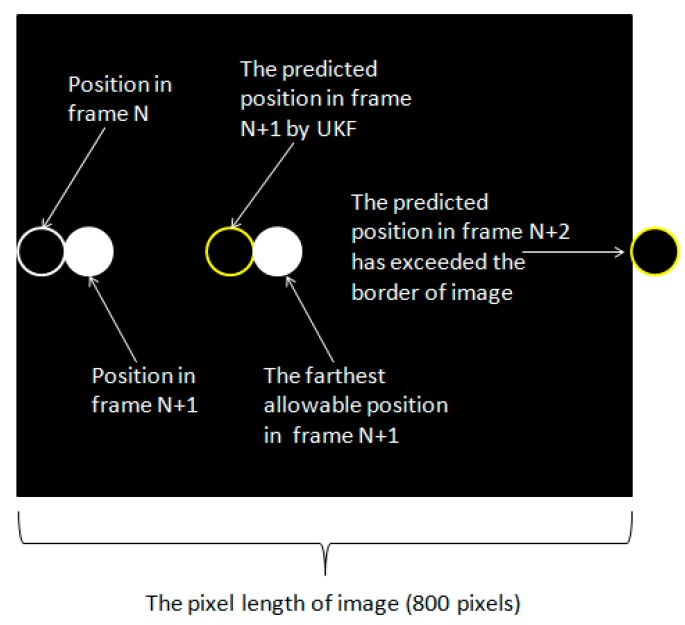
The calculation process of maximum motion speed of the positioning terminal.

To simplify the calculation of the theoretical maximum motion speed of the positioning terminal, we assume that the LED in the image starts moving from the left edge in frame N with no velocity and the motion path is parallel to the x-axis. Because the unscented Kalman filter algorithm can handle variable motion with arbitrary acceleration, we assumed that the UKF algorithm predicts the position according to the uniform acceleration motion model and ignores the effect of the target’s previous positions except its last position. The first maximal moving distance allowed is the diameter of the LED. Then the UKF obtains its speed and accelerated speed to predict its position in the next frame. In frame N + 1, the maximum allowable moving distance is the predicted moving distance plus the diameter of the LED. The steps for the next frame are the same. Then the maximum motion speed can be calculated in frame N + 2, because the predicted moving distance has exceeded the border of image in this frame. The process is shown in Figure 16.

The maximum motion speed of the target is defined as:

(47) $$v_{\max}\;=\;s/t$$

in which s represents the maximum allowable moving distance of the positioning terminal between two successful frames, while t stands for the algorithm’s average processing time. Based on the proportional relationship between images coordinates and world’s coordinates, the relationship can be expressed as:

s/r = D/d(48)

where r represents pixel length of moving distance, D stands for the actual diameter of the LED, and d is the pixel length of the LED. In our experiment, we calculated the average processing time of 87 successful frames without shielding, which is 24.93 ms. By contrast, the algorithm’s average processing time of one frame in our previous work [9] was 0.162 s. The actual diameter of the LED was 150 mm, whose diameter in the image was 60 pixels. Besides, the pixel length of the image was 800 pixels in our experiments.

Also, in our algorithm, if the predicted position of the UKF algorithm exceeded the border, the final output of predicted position will be tangent with the edge of image. Supposing that the LED is tangent with the right edge of the image in frame N + 2, the moving distance is 440 pixels compared with frame N + 1, in other words, r is 440. Therefore, the theoretic maximum allowable motion speed of the positioning terminal is 44.12 m/s ≈ 158.84 km/h according to the definition, which not only meets the requirement of indoor dynamitic positioning, but also satisfies the needs of some outdoor positioning circumstances such as traffic systems in channels. The theoretical maximum allowable motion speed of the positioning terminal in Reference [1] was 48 km/h, much slower than the proposed method.

Robustness is also significant for VLP systems in practical VLP situations, which is often ignored in most existing research. In this article, we especially set-up an experiment measuring the performance of our proposed algorithm under the circumstance of a shielding effect and background interference. Besides, to simplify the analysis, we considered the case that only one LED existed.

**Figure 17:**
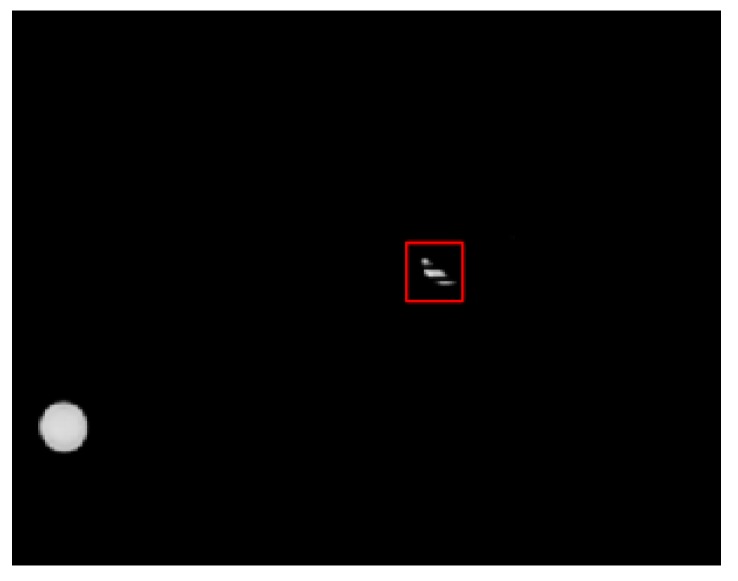
The tracking performance when most of the LED is shielded.

**Figure 18:**
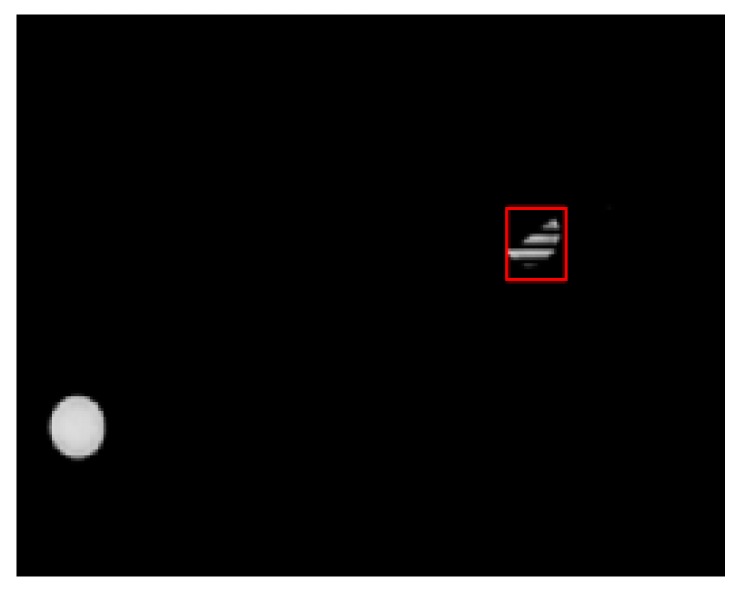
The tracking performance when half of the LED is shielded.

Sometimes LEDs will be shielded or broken, where most existing VLP methods would fail. In contrast, our algorithm realizes high-accuracy positioning under shielded effect by introducing unscented Kalman filter into the mean shift algorithm. The MS has wonderful robustness when the backgrounds are not complicated as shown in Figure 17. However, the algorithm proposed in Reference [9] would lose the LED under the same circumstance. When the LED is shielded, the tracking results of the MS is treated as the observation model of the UKF, combined with the noise matrix to get the final output. Through these measures, the shielded LED can be located with relatively high accuracy and little computational burden. The performance of our proposed algorithm when half the area of the LED is shielded is shown directly in Figure 18. 

**Figure 19:**
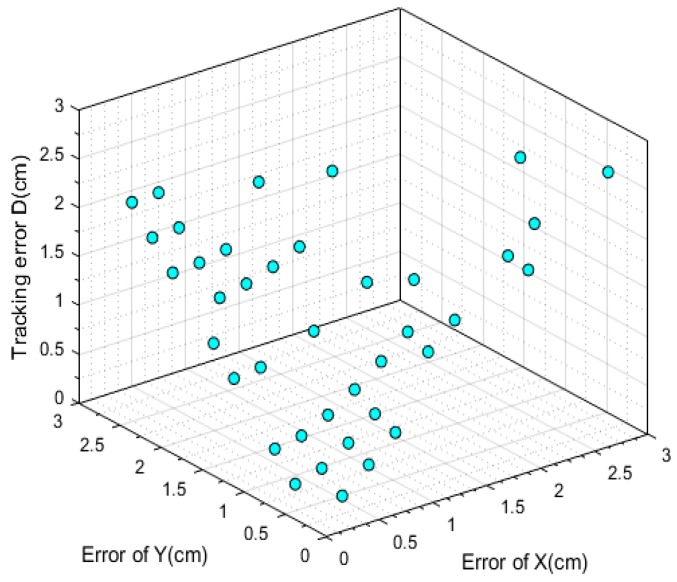
The error under the circumstance where nearly 50 percent of the LED’s area was shielded.

**Figure 20:**
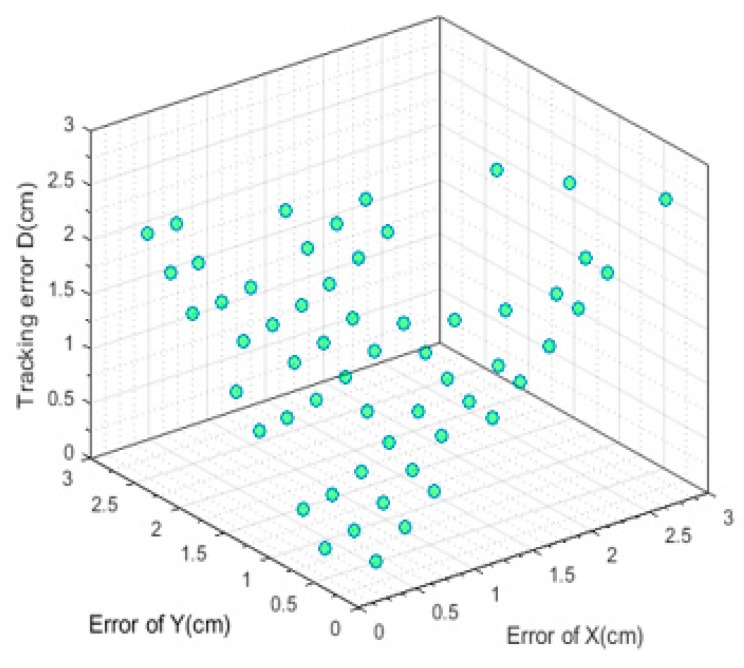
The error under the situation where random areas of the LED was shielded.

In addition, a script was written to make the turlebot3 robot move with the fixed route. Several videos were recorded where the LED was shielded at different degrees and the shielded area varies from 30 percent to 90 percent of the original area. Seventy-four frames where random areas of the LED are shielded and 59 frames where nearly 50 percent of LED’s area was shielded were chosen to analyze the performance of our proposed algorithm handling the circumstances of the shielding effect, whose performances are shown in Figure 19 and Figure 20. 

The average tracking error was 1.41 cm when nearly 50 percent of the LED was shielded. When the LED was shielded randomly, the average positioning error was 1.52 cm. Besides, the average error of the x coordinates in Figure 19 was 0.67 cm and that of the y coordinates was 1.00 cm. In Figure 20, the error of the x coordinates and y coordinates were 0.84 cm and 0.87 cm, respectively. 

**Figure 21:**
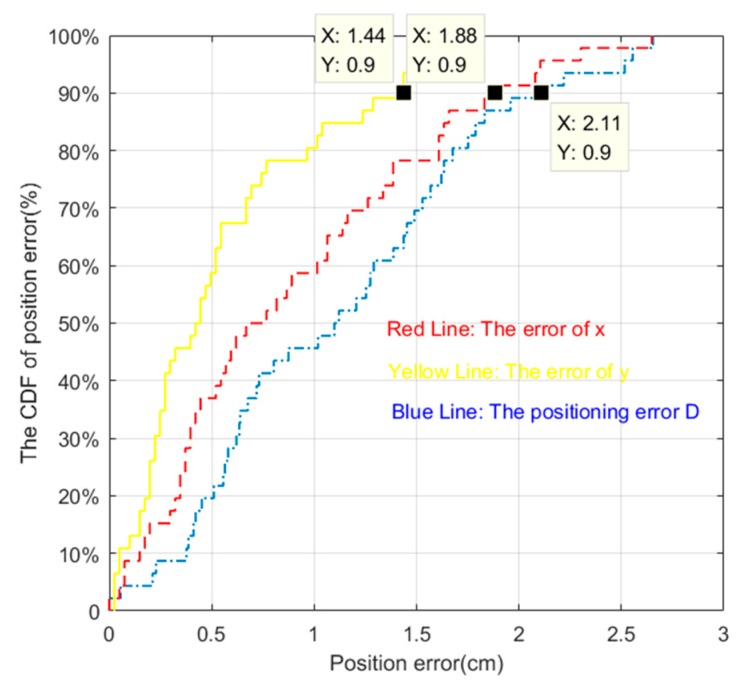
The CDF of positioning error when nearly 50% of the area of the LED was shielded.

**Figure 22:**
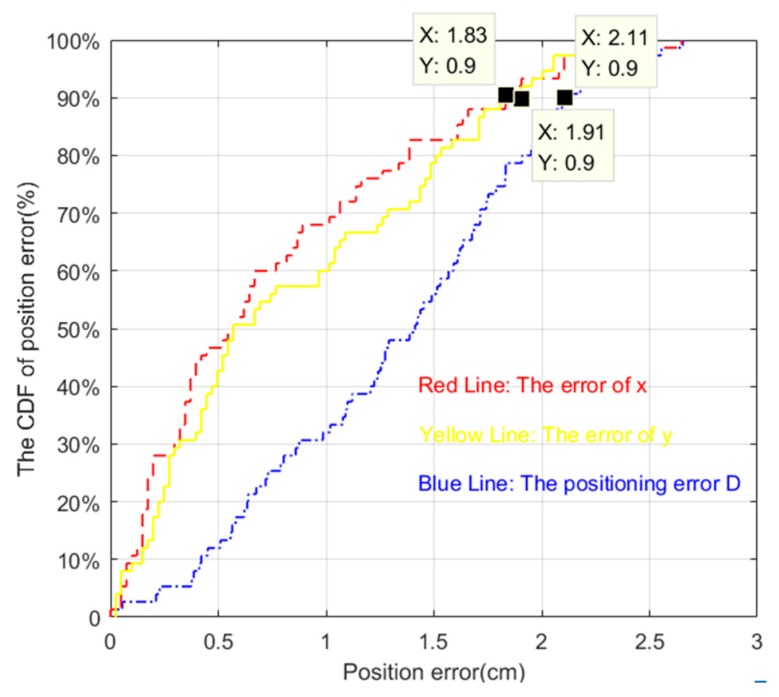
The CDF of positioning error when random areas of the LED was shielded.

The CDF plot of data measuring the position error in Figure 19 and Figure 20 are shown in Figure 21 and Figure 22, respectively. From Figure 21 we can learn that more than 90 percent of tracking error D, the error of x coordinates and y coordinates were less than 2.11 cm, 1.88 cm, and 1.44 cm, respectively when nearly half of the area of the LED was shielded. Meanwhile, these data indexes were 2.11 cm, 1.83 cm, and 1.91 cm, respectively, when random areas of the LED was shielded. These results demonstrate the excellent robustness of our proposed algorithm.

Though the positioning accuracy and the real-time ability of our proposed method did not achieve the most advanced level at present, they are excellent enough compared with most of the VLP systems. Moreover, the strong robustness of our system is unique in the field of VLPs. Before our previous work [9], nobody discussed the aspect of robustness in the field of VLP. Furthermore, the focus of this article is not to improve the real-time ability, robustness or accuracy of the system alone. It is not difficult to improve the performance of any single aspect, but the improvement of them all at the same time and maintaining balance is hard. The key point of our work is balancing the accuracy, robustness, and real-time ability of a VLP system.

## 4. Conclusions

In this paper, we propose a novel VLP algorithm for indoor positioning. The image sensor is used as the positioning terminal and the LED is utilized as the transmitting terminal. The essence and innovation point of our work is replacing the repeating and complex VLP positioning algorithm in a clever way which only needed to know the initial position and relative position relationships of the LED and the positioning terminal. The mean shift algorithm is utilized to track the moving LED in the image sensor, solving the problem of the blur effect and ensuring the real-time ability. The unscented Kalman filter improves the highest allowable motion speed of the positioning terminal as well as reduces the running time of our algorithm. Also, thanks to the excellent robustness of the mean shift algorithm under simple backgrounds, even when most of the LED is shielded in the image, the VLP method will not fail. Furthermore, the results of our visible light positioning method are combined with the noise matrix of UKF to get the output when the LED is shielded, which reduces the error of positioning results. Therefore, our algorithm possesses strong robustness and high accuracy.

As for the experiment, the proposed VLP method can reach a high positioning accuracy up to 0.42 cm and the average processing time per frame is 24.93 ms. Furthermore, even when nearly 50% of the LED is shielded, the positioning accuracy maintains at 1.41 cm, which confirms that our proposed algorithm has strong robustness. All the mentioned results indicate that our proposed VLP method has excellent performance with high positioning accuracy, good real-time ability, and strong robustness. 

## Figures and Tables

**Table 1:** Parameters of experimental facilities and platform.

**Camera Specifications**	
Model	MV-U300
Spectral Response Range/nm	400~1030
Resolution	800 × 600
Frame Rate/FPS	46
Dynamic Range/dB	>61
Signal-to-noise Ratio/dB	43
Pixel(H × V)	2048 × 1536
Pixel Size/μm²	3.2 × 3.2
Time of Exposure/ms	0.0556–683.8
Sensitivity	1.0 V/lux-sec 550 nm
Optical Filter	650nm Low Pass Optical Filter
Type of Shutter	Electronic Rolling Shutter
Acquisition Mode	Successive and Soft Trigger
Working Temperatures/°C	0–50
Support Multiple Visual Software	OpenCV, LabView
**Turtlebot3 Robot Specifications**	
Processor Module	Raspberry Pi 3 B
CPU	Quad Core 1.2 GHz Broadcom BCM2837
	64 bit CPU
RAM	1 GB
operating system	Ubuntu mate 16.04
**Experimental Platform Specifications**	
Size (L × W × H)/ cm^3^	190 × 100 × 190
**LED Specifications**	
Coordinates (x, y, z)/cm	LED1(100,45,190)
	LED2(100,145,190)
	LED3(0,145,190)
	LED4(0,45,190)
The half-power angles of LED/deg(ψ1/2)	60
**Circuit Board Specifications**	
Drive chip	DD311
Drive current/A	0.1
Drive voltage/V	28
